# Lobectomy for Lung Cancer With Partial Anomalous Pulmonary Venous Connection in a Different Lobe: A Case Report

**DOI:** 10.7759/cureus.69672

**Published:** 2024-09-18

**Authors:** Sota Yoshimine, Naohiro Yamamoto, Hisashi Sakano, Yuji Fujita, Norio Akiyama

**Affiliations:** 1 Department of Surgery, Tokuyama Central Hospital, Shunan, JPN

**Keywords:** different lobe, lobectomy, lung cancer, papvc, pulmonary vein

## Abstract

A 78-year-old man was diagnosed with right middle lobe lung cancer, complicated by partial anomalous pulmonary venous connection (PAPVC) in the right upper lobe pulmonary vein. After right middle lobe resection, there was concern about the risk of right heart failure (RHF) due to increased right and left shunting. A pulmonary artery occlusion test using a right heart catheter determined the pulmonary systemic blood flow ratio to be 1.30; the predicted value after the right middle lobectomy was 1.51. The risk of developing RHF after lobectomy was predicted to be low. Therefore, a thoracoscopic right middle lobectomy was performed without PAPVC repair; RHF did not occur postoperatively. Recognizing the presence of PAPVC preoperatively and predicting postoperative hemodynamics when performing lung resection in a patient with PAPVC in the unresected lung are both crucial to avoid fatal postoperative RHF.

## Introduction

Partial anomalous pulmonary venous connection (PAPVC) is a congenital vascular malformation wherein some pulmonary veins directly flow into the systemic circulation veins instead of the left atrium. The prevalence of PAPVC in adults is 0.41%, and owing to the small volume of the left-right shunt, most patients do not have symptoms of right heart failure. Therefore, adult patients with PAPVC are rarely treated; however, caution is required when performing a lung resection [1]. It is rare for patients performing lung resection to also have PAPVC; in cases where PAPVC is localized in the lung to be resected, lung resection also serves as a treatment for PAPVC. Contrastingly, patients with PAPVC in a lobe different from the one resected may require separate PAPVC repair, owing to the increased shunting postresection that may cause right heart failure (RHF) [2]. In such cases, predicting the hemodynamic changes that may occur after lung resection and determining the requirement of PAPVC repair preoperatively is crucial. Herein, we report a case of a patient with right middle lobe lung cancer and PAPVC in the right upper lobe pulmonary vein. The postoperative pulmonary systemic blood flow ratio was predicted using a pulmonary artery occlusion test with a right heart catheter. Thoracoscopic right middle lobectomy was performed without PAPVC repair.

## Case presentation

**Figure 1:**
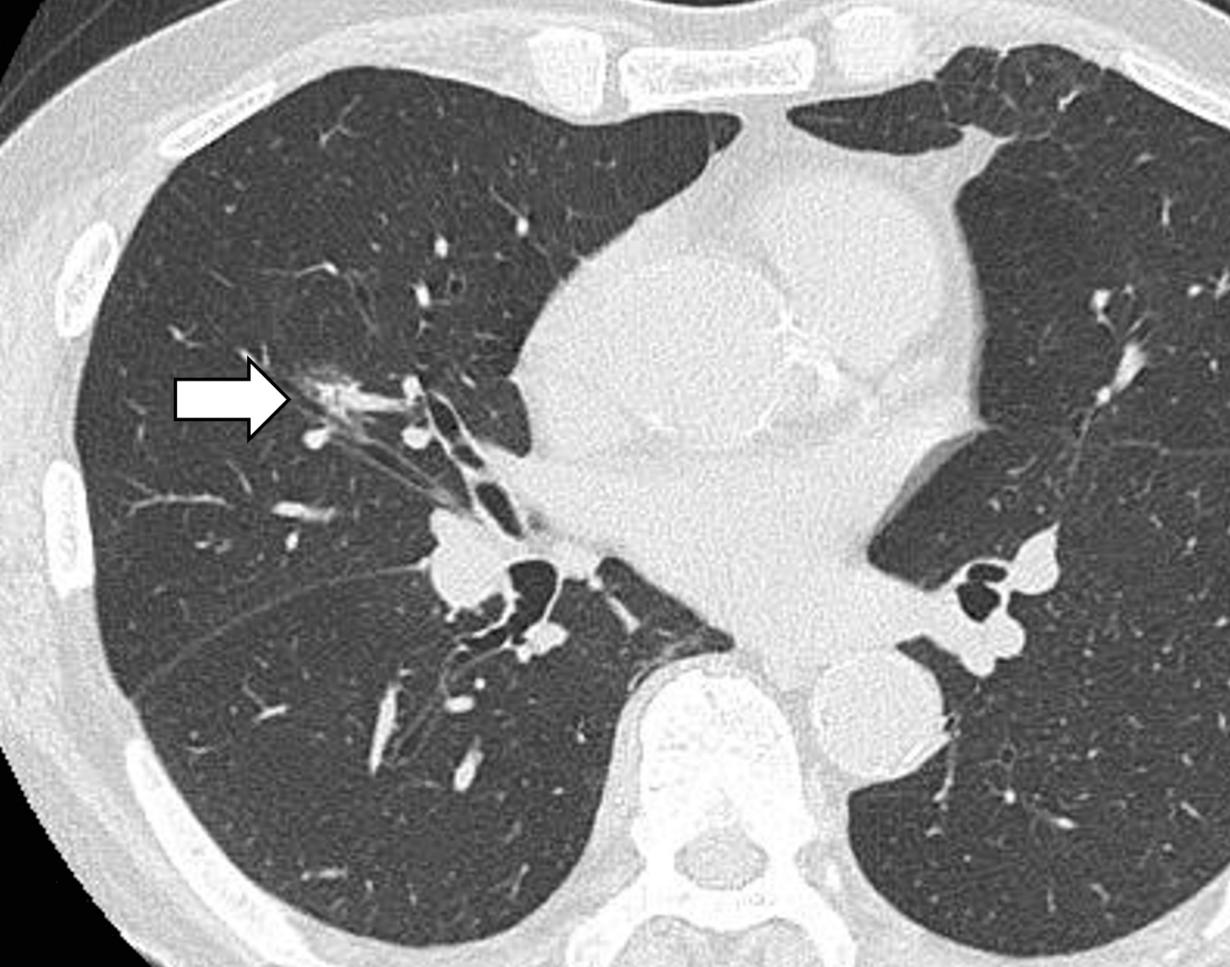
Lung cancer in the right middle lobe.

The patient was a 78-year-old man with hypertension and diabetes mellites. Contrast-enhanced computed tomography (CECT) for health check revealed a partially solid lesion in the right middle lobe (S4) with overall and solid component diameters of 18 mm and 8 mm, respectively (Figure 1).

**Figure 2:**
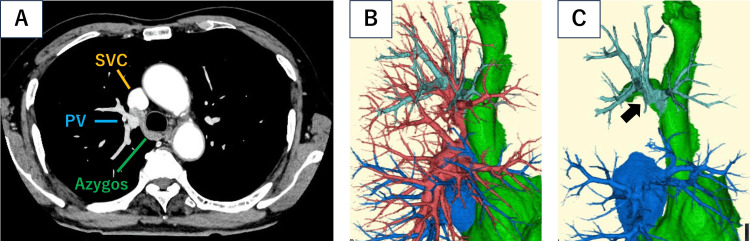
Partial anomalous pulmonary venous connection in the right upper lobe. A: Contrast-enhanced computed tomography (SVC: superior vena cava; PV: pulmonary vein; Azygos: azygos vein) B: Contrast-enhanced computed tomography-based three-dimensional reconstruction (red: pulmonary artery; blue: pulmonary vein; light blue: pulmonary vein of the upper lobe that returns to the vena cava; green: azygos vein and superior vena cava). C: Contrast-enhanced computed tomography-based three-dimensional reconstruction (blue: pulmonary vein; light blue: pulmonary vein of the upper lobe that returns to the vena cava; green: azygos vein and superior vena cava).

Based on systemic examinations, including 18F-fluorodeoxyglucose (FDG)-positron emission tomography (PET), there is no abnormal accumulation in the tumor. The patient was diagnosed with suspected primary lung cancer in the right middle lobe (cT1aN0M0 stage IA1), and radical surgery was planned. A pulmonary function study showed a forced expiratory volume in one second of 89.7% of the predicted volume, a forced vital capacity of 124.1% of the predicted volume, and a carbon monoxide diffusing capacity of 102.4% of the predicted volume. CECT showed that most of the pulmonary veins in the right upper lobe drained into the superior vena cava (SVC) near the confluence of the azygos veins (Fig. 2A, 2B, 2C). 

**Figure 3:**
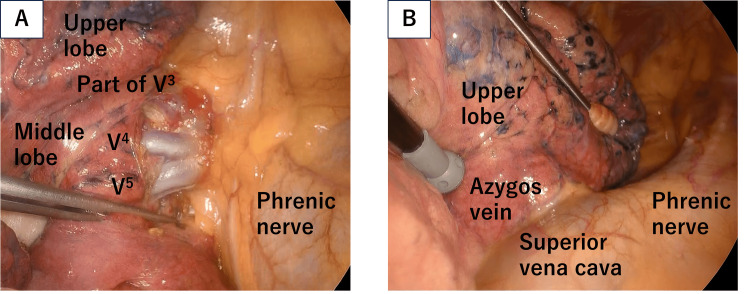
Intraoperative image. A: superior pulmonary vein at the hilum; B: around the superior vena cava

The patient was additionally diagnosed with PAPVC of the right upper lobe pulmonary veins. The patient was asymptomatic, and no abnormal chest auscultation or leg edema was noted. Echocardiography revealed an ejection fraction of 73%; no abnormal wall motion, significant valvular heart disease, or pulmonary hypertension; an estimated pulmonary artery systolic pressure of 23 mmHg was detected; and the estimated pulmonary (Qp) systemic (Qs) blood flow ratio (Qp/Qs) was 1.34. Transesophageal echocardiography revealed no cardiac malformations or atrial septal defects (ASD). Following a discussion between the surgical and cardiovascular teams about the considered risk of an increased left-right shunt after right middle lobectomy, a pulmonary artery occlusion test with right heart catheterization was performed. The initial Qp/Qs increased from 1.30 to 1.51 due to balloon occlusion of A4+5. We determined that the risk of severe RHF is low; a three-port thoracoscopic right middle lobectomy was performed. V4, V5, and part of V3 were observed in the hilum (Figure 3A). The upper lobe was fused near the confluence of the azygos vein and superior vena cava (Figure 3B), assuming that the abnormal vein flowed into the same area. The fissure between the middle and lower lobes was separated using an electric scalpel, and the fissure between the upper and middle lobes was separated using a stapler. A4+5 was resected using a stapler, and V4 and V5 were ligated and cut off, respectively. Lymph nodes #11i and 12m were dissected, the middle lobe bronchus was dissected with a stapler, and the middle lobe was resected. Given the possibility that a right upper lobe pulmonary vein revascularization or lobe resection would be required in the event of postoperative RHF, lymph node #10 and mediastinal lymph node dissections were not performed since the tumor was a ground glass dominant tumor without FDG accumulation on PET.

The postoperative course was uneventful, and the patient was discharged on the seventh postoperative day. The tumor was histopathologically identified as a pT1bN0M0 stage IA2, acinar adenocarcinoma, with overall and infiltration diameters of 2.1 cm and 1.5 cm, respectively. Echocardiography six months postoperatively revealed an ejection fraction of 69%, estimated pulmonary artery systolic pressure was 21 mmHg, and Qp/Qs was 1.26. There was no recurrence of lung cancer or RHF postoperatively.

## Discussion

Patients with PAPVCs in a lobe different from the one resected are at a risk of increased left-right shunt and RHF after lung resection. Black et al. reported a case wherein PAPVC in the contralateral lung was not noticed preoperatively; lung resection led to RHF and eventual death [3]. Therefore, accurate recognition of PAPVC before surgery is important, and CECT-based three-dimensional reconstruction is a valuable tool [1]. PAPVC repair and treatment strategies differ significantly depending on the anatomical context of the abnormal pulmonary vein (ipsilateral or contralateral localization) and its position (left or right) relative to the resected lung [4]. In the case of PAPVC contralateral to the resected lung, it is necessary to repair the PAPVC first and then perform lung resection in two stages [5]. The abnormal pulmonary vein can be connected to the excised pulmonary vein stump, atrium, or atrial appendage on the left side; however, a heart-lung machine is required for revascularization on the right side [2,6]. In our case, it was assumed that it would be necessary to create an artificial ASD and connect the SVC, which had been cut off at a point distal to the inlet of the azygos vein, to the right atrial appendage. Therefore, for an ipsilateral left PAPVC, revascularization may be possible simultaneously with lung resection, even if it is first noticed during surgery. In addition to the highly invasive nature of lung resection and revascularization, complications including stenosis, occlusion, thrombus formation, and arrhythmia can occur following PAPVC repair [6]. Therefore, PAPVC recognition and consideration of the requirement of repair must be carefully performed preoperatively. Qp/Qs, assessed by right heart catheterization, is used as an indicator to determine the requirement of a PAPVC repair; Qp/Qs >1.5-2.0 is considered an indication for PAPVC surgery [7,8]. In cases with PAPVC in the unresected lung, PAPVC repair is not necessary when Qp/Qs = 1.0, while repair is required in symptomatic cases or when Qp/Qs > 1.5. Qp/Qs of 1.0-1.5 represents the borderline range, where the requirement for PAPVC repair is unclear [9]. Qp/Qs may increase after lung resection, and estimation of postoperative Qp/Qs before lung resection is crucial, especially in borderline cases. In the present case, the preoperative and predicted postoperative Qp/Qs in the A4+5 pulmonary artery occlusion test, assuming a right middle lobectomy, were 1.30 and 1.51, respectively. Previous studies have reported cases where the predicted values of postoperative Qp/Qs in the pulmonary artery occlusion test were <1.5, 1.22, and 1.33, respectively; therefore, lung resection was performed without revascularization and RHF did not occur [2,10]. Although the postoperative predicted value in the present case was slightly over 1.5, because right-side PAPVC repair would require highly invasive surgery, revascularization was not performed. Given the possible requirement of surgery in the event of postoperative RHF, the surgical procedures, including lymphoma dissection, were kept to a minimum since the tumor presented as ground glass opacity-dominant and lymph node metastasis was not suspected. Careful long-term follow-up was performed, and postoperative RHF did not occur. The postoperative Qp/Qs at six months had not increased as much as expected, although measurement errors cannot be ruled out. Qp/Qs was calculated using the Doppler method for echocardiography and the Fick method for the pulmonary artery occlusion test. Apart from blood flow through the small pulmonary arteries, veins, and incompetent lobes, changes in the intrathoracic environment due to compensatory expansion of the remaining lung after resection, intrathoracic adhesions, and pleural effusion can cause blood flow changes in the remaining lung. Precise prediction of Qp/Qs in pulmonary artery occlusion tests can be limited. Additionally, the fact that not all pulmonary veins in the upper lobe exhibited PAPVC, coupled with the small volume of the middle lobe resection, could have contributed to the observed lack of exacerbation of postoperative hemodynamics.

## Conclusions

We presented the case of a patient diagnosed with right middle lobe lung cancer, complicated by PAPVC in the right upper lobe pulmonary vein. Pulmonary resection in patients with PAPVC in a lobe different from the resected lung carries the risk of RHF due to an increased left-right shunt. It is crucial to correctly recognize the presence of PAPVC preoperatively and predict hemodynamics postoperatively when performing lung resection in a patient with PAPVC in the unresected lung to avoid fatal postoperative RHF.
